# Combination of Anti-Mycotoxin Additive in Diet Contaminated with Multiple Mycotoxins (Aflatoxin, Fumonisin, Zearalenone and Deoxynivalenol): Effects on Performance and Health of Lambs

**DOI:** 10.3390/ani15192835

**Published:** 2025-09-28

**Authors:** Suelyn de Oliveira Marques, Guilherme Luiz Deolindo, Andrei Lucas Rebelatto Brunetto, Ana Lara Amaral da Veiga, Renato Santos de Jesus, Eduardo Micotti Da Gloria, Gilnei Bruno da Silva, Margarete Dulce Bagatini, Aleksandro Schafer Da Silva

**Affiliations:** 1 Departamento de Zootecnia, Universidade do Estado de Santa Catarina, Chapecó 89815-630, Santa Catarina, Brazil; suelyn.marques@edu.udesc.br (S.d.O.M.);; 2 Programa de Pós-Graduação em Bioquímica e Biologia Molecular, Universidade do Estado de Santa Catarina, Lages 88520-000, Santa Catarina, Brazil; guilhermeluizd@outlook.com (G.L.D.);; 3 Programa de Pós-Graduação em Zootecnia, Universidade do Estado de Santa Catarina, Chapecó 89815-630, Santa Catarina, Brazil; 4 Department of Biological Science, Luiz de Queiroz College of Agriculture, University of São Paulo, Piracicaba 13418-900, São Paulo, Brazil; 5 Centro de Ciências da Saúde, Universidade Federal Fronteira Sul, Chapecó 89812-000, Santa Catarina, Brazil

**Keywords:** sheep farming, mycotoxins, liver injury, oxidative damage

## Abstract

During the grain and cereal production process, whether during harvesting, processing, or storage, errors can occur, compromising product quality and potentially leading to contamination by fungi, which produce toxic substances known as mycotoxins. When fed to animals, these contaminated grains and cereals can cause several negative effects on animal health, impacting their production performance, including immunosuppression, hepatotoxicity, nephrotoxicity, and reproductive problems. To minimize the problems caused by mycotoxins, anti-mycotoxin additives, also known as adsorbents, are used. These are inert materials that bind to mycotoxins and are excreted in feces, preventing their action within the animal’s body. Therefore, the objective of this study was to evaluate the effectiveness of an anti-mycotoxin product based on bentonite, activated charcoal, milk thistle extract, and yeast cell wall in the diet of mycotoxin-contaminated lambs on animal health and performance. Thirty lambs were divided into three homogeneous groups: control (without mycotoxins or additives), mycotoxin (with mycotoxins), and anti-mycotoxin (mycotoxins and additive). The mycotoxins used for feed contamination were aflatoxin (AFLA) (200 ppb), fumonisin (FUMO) (15 ppm), zearalenone (ZEA) (500 ppb), and deoxynivalenol (DON) (1.5 ppm). The anti-mycotoxin additive was used at a dose of 1 kg/ton of concentrate. Parameters of zootechnical performance, hematological profile, serum biochemistry, and oxidative status were evaluated. The group that ingested the contaminated concentrate with mycotoxin had a lower average daily weight gain (ADG) when compared to the control and anti-mycotoxin groups. Ingestion of a mycotoxin-contaminated diet increased the activity of aspartate aminotransferase and gamma-glutamyltransferase, which are indicators of liver damage. However, when the anti-mycotoxin additive was used, the increase in these enzymes was modest and lower than in the mycotoxin group. Ingestion of a mycotoxin-containing concentrate increased levels of oxidative stress biomarkers such as reactive oxygen species (ROS), thiobarbituric acid reactive substances (TBARS), myeloperoxidase (MPO), and reduced glutathione (GST), demonstrating that the mycotoxin challenge was effective in causing oxidative stress. However, when the diet was contaminated with mycotoxins and supplemented with the anti-mycotoxin additive, the levels of ROS and TBARS were similar to those of the negative control group. We concluded that adding the anti-mycotoxin product to the lambs’ diets prevented or minimized the problems caused by mycotoxin consumption, allowing these lambs to have ADG, and feed efficiency similar to the control group.

## Simple Summary

Extreme climates have compromised the production of high-quality cereals or silage, both due to the chemical composition of animal feed and contamination factors, particularly mycotoxins. The problem of mycotoxins, often more than one, has been addressed with the use of anti-mycotoxin additives as a preventative in ruminant diets. In this study, we found that animals exposed to four types of mycotoxins, exceeding the maximum contamination limit recommended by regulatory agencies, interfered with weight gain and in cattle, and increased biomarkers that characterize cell/tissue damage. However, when an additive based on bentonite, activated charcoal, milk thistle extract, and yeast cell wall was added, the negative impacts of these toxins were minimized, with weight gain being similar to that of animals in the control group.

## 1. Introduction

Mycotoxins are toxic chemical compounds produced as secondary metabolites by fungi. They are formed when primary metabolic precursors accumulate. The fungi divert the excess precursors to produce secondary metabolites, known as mycotoxins, thus allowing primary metabolic processes to continue. In Brazil, legislation sets maximum limits only for aflatoxins and zearalenone in animal products [1]. According to Horky et al. [2], there are currently approximately 500 known mycotoxin species, and it is estimated that others have yet to be discovered. Among the most common mycotoxins in feed are deoxynivalenol, zearalenone, ochratoxin, fumonisins, and aflatoxins. These are subdivided into two groups: field mycotoxins, which occur due to fungal contamination before harvest; and storage mycotoxins, which occur due to fungal contamination after harvest. According to Ayalew [3], the fungi involved in the main mycotoxicoses found in animal production are the field fungi *Fusarium graminearum* (deoxynivalenol, nivalenol); *Fusarium moniliforme* (fumonisin); and *Aspergillus flavus* (aflatoxin). Other fungi that colonize the plant before harvest and predispose the product to mycotoxin contamination after harvest, such as *Penicillium verrucosum* (ochratoxin) and *A. flavus* (aflatoxin) [4], indicate that mycotoxins are produced by fungi subjected to specific conditions, such as high humidity, temperature, and flaws in the processing and storage of grains and cereals. Therefore, contamination can vary depending on the environmental conditions and processing methods adopted by each industry. The country’s climate, such as high temperatures and relative humidity, provides optimal growth conditions for pathogenic microorganisms such as fungi.

Mycotoxins are of great importance to human and animal health, where their effects can be acute, with severe symptoms and/or clinical signs observed quickly after ingestion of contaminated feed, or cumulative, causing long-term health effects, such as immunological and carcinogenic effects. Farm animals fed contaminated grains and cereals demonstrate lower productive performance, a consequence of the negative effects of mycotoxins on animal health, which act on the organs responsible for nutrient absorption and digestion. In addition, other effects such as immunosuppression, hepatotoxicity, nephrotoxicity, and reproductive problems are observed [5]. Santurio [6] highlighted that the presence of mycotoxins in feed is subject to environmental factors such as food moisture and ambient temperature. Therefore, mycotoxin contamination can vary depending on environmental conditions, processing methods, production, and storage. Due to the country’s climate, high temperatures, and relative humidity, we have optimal growth conditions for pathogenic microorganisms such as fungi. To minimize the problems caused by mycotoxins, anti-mycotoxin technological additives, also known as adsorbents, are used. These are inert materials that bind to mycotoxins and are excreted in feces, preventing their action within the animal’s body. The anti-mycotoxin blend used in this study is composed of bentonite, activated charcoal, milk thistle extract, and yeast cell wall. This combination of components allows the physical adsorption of mycotoxins through activated charcoal, bentonite, and yeast cell membranes, as well as promoting hepatoprotection through milk thistle extract. Furthermore, the yeast cell membrane also provides protection to the intestinal mucosa.

Milk thistle (*Silybum marianum*) extract has been extensively recognized for its hepatoprotective and antioxidant properties, particularly in mitigating liver damage caused by environmental toxins, such as mycotoxins. Studies have shown that silymarin (the active compound) plays a crucial role in liver cell regeneration, enhancing detoxification processes and reducing oxidative stress induced by toxins in the liver [7,8]. Thus, the addition of this extract in the anti-mycotoxin formulation is intended to support liver health, which is essential for mitigating the adverse effects of mycotoxins on animals.

The inclusion of yeast cell wall in this formulation is based on its dual function as both an adsorbent and a protective agent. Rich in mannans and β-glucans, the yeast cell wall can effectively bind to both polar and non-polar mycotoxins, preventing their absorption into the bloodstream. Moreover, it contributes to the protection of the intestinal mucosa, which is essential for reducing the inflammatory effects of mycotoxins on the gastrointestinal system [9]. Therefore, its inclusion in the formulation offers a multifaceted approach to mitigating mycotoxin toxicity.

Therefore, the hypothesis of this study is that the anti-mycotoxin blend, which contains components and ingredients with adsorbent properties, blocks and minimizes the effects of mycotoxins present in animal feed, thus improving animal health, and production performance. The aim of this study is to evaluate whether adding an anti-mycotoxin additive formulated with bentonite, activated charcoal, milk thistle extract, and yeast cell membranes to diets contaminated with multiple mycotoxins (aflatoxin, fumonisin, deoxynivalenol, and zearalenone) can minimize the negative impacts of these toxins on lamb performance and health.

## 2. Materials and Methods

The experiment was conducted in the sheep farming sector of the Experimental Farm of the Centro de Educação Superior do Oeste (FECEO), located in the city of Guatambu/SC, situated at latitude −27°9′6.513″ S, and longitude 52°47′19.468″ W.

### 2.1. Additive Characterization

The anti-mycotoxin additive used in this study is composed of bentonite, activated charcoal, milk thistle extract, and yeast cell wall; a product currently in the registration phase from Tectron. The additive was used at a dose of 1 kg per ton of concentrate, as recommended by the manufacturer.

### 2.2. Mycotoxin Inoculum Production and Challenge Dose

The mycotoxins used in this experiment were produced by Micotech using isolated fungi cultivated on rice and corn. Aflatoxins were produced by an *Aspergillus nomius* isolate, fumonisins by a *Fusarium verticilloides* isolate, and deoxynivalenol and zearalenone by a *Fusarium graminearum* isolate. Fermentation was carried out in 500 mL Erlenmeyer flasks, to which 100 g of rice or corn were added. The material was moistened with 40 mL of distilled water and autoclaved at 121 °C for 30 min (CS-75, Prismalab, Rio de Janeiro, RJ, Brazil). The flasks were then left at room temperature. The autoclaved material was inoculated with 2 mL of conidial suspension of each fungus (1 × 10^8^ conidia per mL). The conidial suspension was obtained from colonies of each fungus cultivated on potato dextrose agar for 10 days at 25 °C. After inoculation, the vials were kept static for 21 days at a controlled temperature (25 °C). Subsequently, the fermented material was dried in an oven at 57 °C and ground in a mill with a sieve < 0.85 mm to be used to artificially contaminate the feed. The challenge-dose stipulated for this study had a contamination level in the diet of approximately 200 ppb of aflatoxins (169.6 g per 100 kg of concentrate), 15 ppm of fumonisins (621.0 g per 100 kg of concentrate), 1.5 ppm of deoxynivalenol (1077.0 g per 100 kg of concentrate), with the addition of this amount of deoxynivalenol consequently the contamination level of zearalenone was 500 ppb. The levels used were determined based on data from the pilot study that showed concentrations near or above the tolerance limits set by regulatory agencies, sufficient to induce subclinical effects (unpublished data). In the literature, information on mycotoxin concentrations and clinical signs when four or more are involved is limited, which is why a pilot study was necessary.

### 2.3. Animals and Installation

Thirty castrated male Lacaune lambs with an average initial body weight of 20 ± 1.4 kg, and an average age of 60 days were used. The animals were preventively dewormed with the anthelmintic levamisole hydrochloride—5% (Ripercol^®^, Zoetis, Brazil) at the manufacturer’s recommended dosages. The animals were housed in individual pens measuring 1.0 m × 2.0 m with cement floors and individual access to feed and water. At 60 days of age, the lambs had a functional rumen, though still in the maturation process. This age and weight were chosen to assess the vulnerability of this age group to mycotoxin effects, as lambs are typically more susceptible to these toxins than adult animals. The project was submitted to the Animal Use Ethics Committee (CEUA) of the State University of Santa Catarina (UDESC) and approved under protocol no. 5513260824.

### 2.4. Experimental Design

The experimental design was completely randomized, with three treatments and 10 replicates, with each lamb representing an experimental unit. The experiment lasted 48 days, including a 15-day adaptation period to the stalls, management, and feeding (diet without mycotoxin and additive). Following this, the 33-day experimental period began. Three treatments were evaluated: control group (no mycotoxin or anti-mycotoxin); mycotoxin group (with the addition of a mycotoxin mixture); and anti-mycotoxin group (with the addition of mycotoxins and anti-mycotoxins).

### 2.5. Experimental Diet

The experimental diets (Table 1) were formulated according to the animals’ nutritional requirements, following the Nutrient Requirements of Small Ruminants [10]. They were isoprotein and isoenergetic, and fed to the lambs twice daily (8:00 a.m. and 4:00 p.m.). The concentrate was offered to the lambs first, and only after complete consumption (a 25 min period) was corn silage provided. This methodology was adopted to ensure maximum total consumption of both the mycotoxin and the additive. The lambs had free access to food and water. The concentrates of the mycotoxin and anti-mycotoxin groups were artificially contaminated with the challenge dose, and the adsorbent material was also added to the anti-mycotoxin group.

The amount of feed provided and the remaining feed were recorded for each animal. Samples of concentrate and corn silage were collected, placed in plastic bags, and frozen (−20 °C) for analysis to determine their chemical composition. The concentrate and corn silage samples were analyzed for dry matter (DM), mineral matter (MM), crude protein (CP), ether extract (EE), neutral detergent fiber (NDF) and acid detergent fiber (ADF) were determined. The ingredients and chemical composition of the diets are shown in Table 1 and Table 2, respectively.

### 2.6. Animal Performance

To monitor animal performance, we weighed the animal’s biweekly at four different times (arrival, day 1, 15, and 30) using a digital scale, while the animals were fasting. Average daily gain (ADG) was calculated by the ratio of weight gain to the number of days in confinement, while feed conversion was calculated by the ratio of total feed intake to weight gain during the confinement period.

### 2.7. Hematological Profile and Serum Biochemistry

Blood samples were collected at three points: on days 14, 28, and 48, by puncture of the jugular vein of fasting animals. Samples were transported refrigerated using vacuolated EDTA tubes without anticoagulant. Hemoglobin, erythrocyte count, total leukocyte count, hematocrit, and leukocyte differentiation were determined immediately upon arrival at the laboratory using an automated hematology analyzer (3-part EQUIP VET 3000^®^, Barueri, Brazil).

The tubes without anticoagulant were centrifuged at 7000 rpm for 10 min to separate the serum for biochemical analysis. The supernatant was transferred to 1.5 mL microtubes, labeled, and stored at −20 °C until analysis. Serum levels of total protein (PT), glucose, albumin, ferritin, creatine kinase (CK-NAC), cholinesterase, cholesterol, triglycerides, alanine aminotransferase (ALT), gamma-glutamyltransferase (GGT), aspartate aminotransferase (AST), and urea were analyzed using an automatic analyzer (Zybio^®^ EXC 200, Barueri, Brazil), and commercial kits (Analisa^®^, Gold Analisa Diagnóstica Ltda, Belo Horizonte, Brazil). Globulin levels were obtained through mathematical calculation (total protein—albumin).

### 2.8. Oxidative Status Biomarkers

From the tubes without anticoagulant, serum was extracted by centrifugation to measure lipid peroxidation by the amount of thiobarbituric acid reactive substances (TBARS), using the method of Jentzsch et al. [11]. To determine reactive oxygen species (ROS), the technique described by Ali et al. [12] was applied. The determination of reduced glutathione (GSH) was performed according to the method described by Tietze [13]. To determine protein thiols (PSH), the technique described by Ellman [14] was applied.

### 2.9. Mycotoxin Quantification in Diets

For mycotoxins analyses samples of feed were ground to <0.85 mm and one gram of the ground material was transferred to test tube of 50 mL according to the methodology described by Santos et al. [15]. Detection and quantification of mycotoxins were performed with high-performance liquid chromatography coupled with tandem mass-spectrometry (LC/MS/MS) [15]. The mass-spectrometer was operated in scheduled multiple reaction monitoring (MRM) in positive mode. The data acquisition of mass spectrometer are showed in Table S1. Mycotoxins quantification was carried out using matrix-matched calibration curves, using extracts of diets phases not contaminated. The final contamination level observed in the contaminated diets is presented in Table S2.

### 2.10. Statistical Analysis

The data showed normal distribution after being subjected to the Shapiro–Wilk test; skewness, kurtosis, and homogeneity were assessed using Levene’s test; linearity was assessed using linear regression. Based on these previous results, our data were analyzed using the SAS MIXED procedure (SAS Inst. Inc., Cary, NC, USA; version 9.4) to determine the denominator degrees of freedom for the fixed effects test (day, treatment, and treatment × day interaction) in a completely randomized design to determine ADG and feed efficiency. All other variables (body weight, feed intake, serum chemistry, and complete blood count) were analyzed as repeated measures and tested for fixed effects of treatment, day, and treatment × day (group) interaction, and animal as random variables. All results obtained on d14 for each variable were also included as covariates, as was initial body weight; however, the command for covariates was removed from the model when *p* > 0.05. Mean comparison analysis was performed using the Tukey test. All results were expressed as mean and standard error (SEM), with significance considered when *p* ≤ 0.05.

## 3. Results

### 3.1. Performance

The performance results are described in Table 3. There was no difference between the groups for body weight (*p* > 0.05); as well as no difference was observed for feed intake (*p* > 0.05). There was a significant difference in average daily weight gain (*p* = 0.01), being 10% lower in the lambs of the mycotoxin group when compared to the other groups. Similarly, feed efficiency was significantly 10.7% lower in the lambs of the mycotoxin group when compared to the control.

### 3.2. Hematological Profile

The hematologic results are described in Table 4. There was no treatment × day interaction for red and white blood cell series (*p* > 0.05); but there was a treatment effect for lymphocyte (*p* = 0.05) and granulocyte (*p* = 0.02) counts. Both groups of lambs that consumed mycotoxin (anti-mycotoxin and mycotoxin) had lower lymphocyte counts compared to the control group; the granulocyte count was only lower in the lambs in the anti-mycotoxin group when compared to the control. There was no difference between groups for the variables total leukocytes, monocytes, erythrocytes, hemoglobin, hematocrit, and platelets.

### 3.3. Seric Biochemistry

The results of serum analysis related to metabolism, function, and liver and inflammatory injury are described in Table 5. No difference was observed between groups for albumin, total protein, and globulin levels (*p* > 0.05). Creatine kinase activity was higher in the serum of lambs in the mycotoxin group when compared to the control (*p* = 0.01) on days 28 and 48. There was a difference between groups for cholesterol (*p* = 0.05) and triglyceride (*p* = 0.04) levels, with cholesterol being higher in lambs in the mycotoxin group compared to the anti-mycotoxin group; triglyceride levels were higher in the blood of lambs in the mycotoxin group when compared to the other groups. There was no difference between groups for ferritin, glucose, urea, and bilirubin levels, as well as cholinesterase and ALT activities (*p* > 0.05). GGT and AST activity were affected by treatment, that is, mycotoxin ingestion, which increased the activity of these enzymes. GGT activity was higher in lambs in the mycotoxin (↑ 39.9%) and anti-mycotoxin (↑ 24.4%) groups compared to the control on day 48 of the experiment. AST activity on days 28 and 48 was higher in the blood of the mycotoxin group compared to the other two groups; however, on day 48, we also found that AST activity was higher in the anti-mycotoxin group compared to the control, but significantly lower than in the blood of lambs in the mycotoxin group.

### 3.4. Oxidative Status

The levels of oxidative stress biomarkers are presented in Table 6. There was no difference between groups for PSH (non-enzymatic antioxidant) levels. Only on day 48 was a significant difference observed for ROS and TBARS levels (*p* = 0.01) and MPO activity (*p* = 0.03), being higher in the serum of lambs in the mycotoxin group when compared to the other groups. There was a difference between groups for GSH levels (*p* = 0.05), i.e., on days 28 and 48, these levels were higher in the blood of the control and anti-mycotoxin groups when compared to the mycotoxin group.

## 4. Discussion

Average daily gain and feed efficiency were influenced by the mycotoxin-contaminated diet, as these caused health problems and, consequently, negatively impacted performance. No significant differences were observed in the final body weight of the lambs. This result can be attributed to the relatively short experimental period (48 days). A longer exposure time may have allowed the differences in performance to become more evident. Furthermore, the similarity in initial and final body weights of the lambs may have contributed to the lack of statistical significance in final body weight, as the small initial weight variations limited the potential for growth differences during the study. Ruminants are less affected by mycotoxins than no-ruminants due to their ruminal microbiota, as mycotoxins can be degraded and/or bio converted in the rumen by microorganisms [16,17,18]. This explains the lack of significant data on body weight, and feed intake.

Although the lambs used in this study were 60 days old, they already had a functional rumen, though still in the maturation process. This age and weight were chosen intentionally to assess the vulnerability of this age group to the effects of mycotoxins, as lambs can be more susceptible to these toxins than adult animals. Although ruminal microorganisms have the potential to mitigate the toxic effects of mycotoxins [16,17], the ability of the rumen in younger animals to perform this function efficiently could be a limitation.

In comparison with most commercial products, which are usually composed only of clay minerals or activated charcoal with a predominant adsorptive action, the formulation tested in this study combines components with complementary functions. Bentonite and activated charcoal provide high binding capacity for different mycotoxins due to their porous structure and large surface area [19,20]. Yeast cell wall, rich in mannans and β-glucans, contributes both to the physical adsorption of polar and non-polar mycotoxins and to the protection of the intestinal mucosa [9]. In addition, milk thistle (*Silybum marianum*) extract is well recognized for its hepatoprotective and antioxidant properties, supporting cell regeneration and reducing oxidative stress induced by mycotoxins [7,8]. This combination allowed not only the reduction in intestinal absorption of mycotoxins but also provided additional support to hepatic integrity and oxidative balance, aspects that are still poorly explored in traditional commercial formulations.

Hematology and blood profile data were used as indicators of the health status of the experimental animals; mild immunosuppression was observed when the lambs consumed the mycotoxin-containing diet. This is because one of the main functions of lymphocytes is the formation of antibodies in response to antigens present in the body [21]. In this study, lymphocytes and granulocytes showed variations in the groups that consumed the mycotoxins (anti-mycotoxin and mycotoxin groups), indicating a negative effect of mycotoxin intake on the cellular immune system, compromising the body’s efficient immune response. Falkauskas et al. [22] highlight that DON inhibits protein synthesis, with the cells most affected being those that actively produce proteins: lymphocytes and the liver. Researchers indicated that in their preliminary experiments, DON exposure induces the overexpression of cytokines and chemokines, leading to immunological stress, which caused damage to immune function, indicating a suppressed immune system [23,24].

Edrington et al. [25] and Xiong et al. [26] highlight GGT, AST, ALT, and ALP as indicators of liver function; therefore, changes in these parameters indicate possible liver injury. As expected, two variables were affected by mycotoxin ingestion: GGT and AST, that is, both increased their activity. GGT is one of the enzymes that reflect liver function in ruminants [26,27], and elevated AST activity has been reported to be associated with liver damage [28]. Mycotoxins influence liver function, but their effects vary according to the dose and duration of the toxin, being cumulative over time. In this study, GGT and AST activity were affected by treatment;, i.e., mycotoxin ingestion increased the activity of these enzymes, suggesting that the contaminated diet caused some degree of liver injury. In the 1990s, Edrington et al. [25] reported, in a study conducted with growing lambs, the damage caused by aflatoxins to liver and hepatobiliary cell function, with an increase in both serum GGT and AST activity, similar to what we observed in our study.

Creatine kinase is an intracellular enzyme present primarily in skeletal muscle, myocardium, and brain, and in small amounts in visceral tissues. Therefore, elevated serum CK levels indicate possible damage to these sites, as rupture of cell membranes releases CK from the cytosol into the circulation. In this study, significant values were observed for animals exposed to mycotoxins (anti-mycotoxin and mycotoxin groups), while lower values were observed for the anti-mycotoxin group, indicating that the use of the adsorbent additive was effective in mitigating the damage caused by the toxins. The indications of liver damage justify the difference between groups in cholesterol (*p* = 0.05) and triglyceride (*p* = 0.04) levels, with cholesterol being higher in lambs in the mycotoxin group compared to the anti-mycotoxin group. We believe that liver dysfunction, which causes difficulty storing cholesterol in the liver, was responsible for the failure to metabolize and store fat, which remained free in the circulation, a fact that could explain the increase in these levels biomarkers.

Oxidative stress arises from an imbalance between oxidants and the antioxidant enzyme system, which causes the oxidation of biomolecules and the consequent loss of their biological functions, leading to potential oxidative damage to cells and tissues [29]. Huang et al. [30] tested mycotoxin contamination in dairy goat diets and reported an intense oxidative burst, indicating mycotoxins as important inducers of oxidative stress. Contamination in ruminants impacts cellular homeostasis; moreover, the liver, a crucial organ for the body’s functioning, is also affected. If liver function is compromised, the activity of several enzymes is impaired, particularly AST and ALT. This is because increased AST activity is linked to oxidative activity, where these negative effects lead to the production of free radicals. This study demonstrated the cumulative effects of mycotoxin exposure, with elevated ROS and TBARS levels, both indicators of oxidation and cell damage, observed on day 48. A significant difference was observed between groups for GSH levels (*p* = 0.05), with GSH activity being higher in the blood of the control and anti-mycotoxin groups on days 28 and 48 compared to the mycotoxin group. This indicates that oxidative stress was greater in animals that consumed the contaminated concentrate without the adsorbent, demonstrating that the additive was effective in mitigating the damage caused by mycotoxins. Dasari [31] reports that aflatoxicosis decreased GSH concentrations and GST and GR activities, suggesting an inefficiency in the formation of ROS and an inability to detoxify xenobiotic cells (GSTs).

Myeloperoxidase activity was higher in the serum of lambs in the mycotoxin group compared to the other groups at the end of the experiment. The MPO enzyme converts hydrogen peroxide (H_2_O_2_) into hypochlorous acid, the latter of which is highly effective in killing pathogenic invaders, such as bacteria, but also potentially causes cytotoxic damage to host tissue [32]. When the antioxidant system is compromised, negative effects on the activity of the MPO enzyme, which is essential for the pro-inflammatory response, are observed. Therefore, the anti-mycotoxin additive was effective in reducing MPO levels when compared to the group that consumed the contaminated concentrate without the anti-mycotoxin additive.

## 5. Conclusions

Ingestion of mycotoxin-contaminated concentrate impaired average daily weight gain and caused liver damage, as well as elevated levels of oxidative stress biomarkers, demonstrating that the mycotoxin challenge occurred and enabling the validation of this study. Adding the anti-mycotoxin product to the lambs’ diets prevented or minimized the problems caused by mycotoxin consumption, allowing these lambs to have ADG and feed efficiency similar to the control group, as well as lower levels of oxidative markers and liver damage.

## Figures and Tables

**Table 1:** Ingredients of the experimental diet used in this research.

Ingredients	Composition (g/kg)
Corn silage	0.430
Ground corn	0.350
Soybean meal	0.150
Wheat bran	0.120
Mineral and vitamin premix	0.030
Limestone	0.010

Calcium (Min.) 160.00 g/kg; calcium (Max.) 232.00 g/kg; sulfur (Min.) 20.00 g/kg; phosphorus (Min.) 30.00 g/kg; magnesium (Min.) 24.00 g/kg; potassium (Min.) 25.00 g/kg; sodium (Min.) 48.00 g/kg; cobalt (Min.) 20.00 mg/kg; organic chromium (Min.) 6.00 mg/kg; iron (Min.) 450.00 mg/kg; iodine (Min.) 26.00 mg/kg; manganese (Min.) 660.00 mg/kg; selenium (Min.) 6.50 mg/kg; zinc (Min.) 805.00 mg/kg; vitamin A (Min.) 100,000.00 IU/kg; vitamin D3 (Min.) 30,000.00 IU/kg; vitamin E (Min.) 300.00 IU/kg; *Saccharomyces cerevisiae* (Min.) 25,000,000,000.00 CFU/kg.

**Table 2:** Chemical composition of feeds in the experimental diet.

Chemical Composition (%) ^1^	Corn Silage	Concentrate Control	Concentrate Anti-Mycotoxin	Concentrate Mycotoxin
Dry matter (DM), %	37.71	96.48	96.57	96.05
Crude protein, %	9.14	16.87	17.11	17.50
Ethereal extract, %	4.28	3.40	3.33	3.30
Ashes, %	6.07	7.89	7.70	7.14
NDF, %	44.56	13.50	12.35	13.15
ADF, %	20.74	6.02	5.52	6.19

^1^ Results based on DM.

**Table 3:** Body weight, average daily gain, feed intake and feed efficiency of lambs challenged with a diet contaminated with mycotoxins in the presence of an anti-mycotoxin technological additive.

Variables	Control	Anti-Mycotoxin	Mycotoxin	SEM	*p*: Treat ^3^	*p*: Treat × Day ^4^
Body weight (kg)					0.34	0.21
d1 ^1^	20.3	20.2	20.6	0.45		
d14 ^2^	22.7	22.8	22.9	0.42		
d28	26.1	26.4	25.9	0.41		
d48	31.6	31.7	30.9	0.41		
ADG (kg)						
d14–48	0.261 ^a^	0.261 ^a^	0.235 ^b^	0.01	0.01	NE
DMI (kg DM)						
d14–48	1.07	1.11	1.08	0.03	0.83	0.94
Feed efficiency (kg/kg)						
d14–48	0.243 ^a^	0.235 ^ab^	0.217 ^b^	0.006	0.03	NE

^1^ Beginning of the adaptation period; when the feed was not yet contaminated with mycotoxins (duration of this period: 14 days). ^2^ Beginning of the experimental period, when the lambs’ diet was contaminated with mycotoxins and fed to the animals for 34 days (d14-d48). ^3^ When *p* ≤ 0.05, the treatment effect was considered significant, illustrated by different letters on the same line. ^4^ No treatment × day interaction was observed for body weight and feed intake, and the other variables were not evaluated (NE).

**Table 4:** Blood count of lambs challenged with a diet contaminated with mycotoxins in the presence of anti-mycotoxin technological additive.

Variables	Control	Anti-Mycotoxin	Mycotoxin	SEM	*p*: Treat ^1^	*p*: Treat × Day ^2^
Leukocytes (×10^3^/µL)	8.79	7.29	7.39	0.50	0.15	0.32
Lymphocytes (×10^3^/µL)	5.82 ^a^	4.89 ^b^	4.93 ^b^	0.19	0.05	0.26
Granulocytes (×10^3^/µL)	1.65 ^a^	1.43 ^b^	1.50 ^ab^	0.08	0.02	0.11
Monocytes (×10^3^/µL)	1.31	0.96	0.94	0.11	0.41	0.58
Erythrocytes (×10^6^/µL)	10.1	10.2	9.71	0.10	0.97	0.96
Hemoglobin (mg/dL)	12.0	11.9	11.3	0.12	0.92	0.95
Hematocrit (%)	29.2	29.4	27.9	0.32	0.73	0.46
Platelets (×10^3^/µL)	216	215	251	12.2	0.15	0.23

^1^ When *p* ≤ 0.05, the treatment effect was considered significant, illustrated by different letters on the same line. ^2^ No treatment × day interaction was observed for these hematologic variables.

**Table 5:** Serum biochemistry of lambs challenged with a diet contaminated with mycotoxins in the presence of an anti-mycotoxin technological additive.

Variables	Control	Anti-Mycotoxin	Mycotoxin	SEM	*p*: Treat ^1^	*p*: Treat × Day ^2^
Albumin (g/dL)	3.18	3.24	3.17	0.04	0.79	0.65
Total protein (g/dL)	5.81	6.12	5.83	0.08	0.66	0.59
Globulin (g/dL)	2.63	2.88	2.66	0.05	0.87	0.92
Creatine kinase (U/L)					0.01	0.01
d14	568	595	578	79.3		
d28	394 ^b^	470 ^b^	747 ^a^	76.1		
d48	194 ^b^	309 ^ab^	326 ^a^	41.5		
Mean	294 ^b^	395 ^ab^	537 ^a^	62.4		
Cholesterol (mg/dL)	77.0 ^ab^	74.5 ^b^	82.1 ^a^	2.36	0.05	0.11
Triglycerides (mg/dL)	31.7 ^b^	32.4 ^b^	37.8 ^a^	1.78	0.04	0.12
Cholinesterase (U/L)	182	172	183	8.41	0.55	0.72
Ferritin (µg/L)	255	252	250	6.21	0.86	0.77
GGT (U/L)					0.01	0.01
d14	60.1	60.3	63.1	2.74		
d28	55.8	64.7	66.1	3.25		
d48	53.1 ^c^	66.1 ^b^	74.3 ^a^	2.19		
Mean	54.4 ^b^	65.4 ^ab^	70.2 ^a^	2.56		
AST (U/L)					0.01	0.01
d14	91.2	89.1	94.2	2.68		
d28	90 ^b^	90.1 ^b^	105 ^a^	2.74		
d48	92.2 ^c^	104 ^b^	115 ^a^	2.98		
Mean	91.6 ^c^	98.5 ^b^	110 ^a^	2.19		
ALT (U/L)	20.1	20.9	22.1	0.69	0.23	0.15
Glucose (mg/dL)	78.4	83.2	80.9	1.41	0.36	0.52
Urea (mg/dL)	48.3	46.6	45.9	1.21	0.75	0.64
Bilirubin (mg/dL)	0.045	0.046	0.046	0.002	0.98	0.94

^1^ When *p* ≤ 0.05, the treatment effect was considered significant, illustrated by different letters on the same line. ^2^ When *p* ≤ 0.05, the treatment × day interaction was considered, illustrated by different letters on the same line.

**Table 6:** Oxidative status of lambs challenged with a diet contaminated with mycotoxins in the presence of an anti-mycotoxin technological additive.

Variables	Control	Anti-Mycotoxin	Mycotoxin	SEM	*p*: Treat ^1^	*p*: Treat × Day ^2^
PSH (µmol/L)	4.61	4.01	4.27	0.28	0.91	0.83
ROS (Fluorescence)					0.68	0.01
d14	14.8	16.6	15.9	1.91		
d28	12.2	10.4	10.7	0.58		
d48	12.9 ^b^	12.1 ^b^	18.1 ^a^	0.94		
Mean	12.6	11.2	14.4	0.76		
TBARS (nmol/mL)					0.35	0.01
d14	58.3	57.4	56.9	0.75		
d28	59.6	61.5	63.8	5.07		
d48	73.8 ^b^	72.2 ^b^	87.5 ^a^	2.01		
Mean	66.7	66.8	75.6	2.31		
MPO (µM of quinoneimine/30 min)					0.63	0.03
d14	4.74	5.51	4.48	0.55		
d28	2.92	2.29	2.61	0.35		
d48	3.03 ^b^	2.21 ^b^	5.88 ^b^	0.34		
Mean	2.97	2.25	4.24	0.35		
GSH (mmol/g protein)					0.05	0.03
d14	28.8	30.2	30.6	0.56		
d28	30.7 ^ab^	33.2 ^a^	27.3 ^b^	0.77		
d48	32.5 ^a^	30.1 ^ab^	28.2 ^b^	0.78		
Mean	31.6 ^a^	31.6 ^a^	27.7 ^b^	0.64		

^1^ When *p* ≤ 0.05, the treatment effect was considered significant, illustrated by different letters on the same line. ^2^ When *p* ≤ 0.05, the treatment × day interaction was considered, illustrated by different letters on the same line.

## Data Availability

Data can be made available upon request.

## Supplementary Materials

The following supporting information can be downloaded at: https://www.mdpi.com/article/10.3390/ani15192835/s1, Table S1. Acquisition parameters data from mass spectrometer. Table S2. Mycotoxin contamination in the experimental diet of lambs.
