# Supplementary material for: Combination of Anti-Mycotoxin Additive in Diet Contaminated with Multiple Mycotoxins (Aflatoxin, Fumonisin, Zearalenone and Deoxynivalenol): Effects on Performance and Health of Lambs
Source: Animals (Basel). 2025 Sep 28;15(19):2835. doi: 10.3390/ani15192835 (PMC12523754; doi:10.3390/ani15192835)
Supplement: Supplementary file 1 [file animals-15-02835-s001.zip › animals-3886109-supplementary.pdf]

animals

MDPI

Table S1. Acquisition parameters data from mass spectrometer.

Analyte	MRM Transiction	Dwell Time (s)	Cone Voltage (V)	Collision Energy (eV)
Aflatoxin B1	313.08>241.23	0.005	30	37
	313.08>285.39			23
IS Aflatoxin B1	330.00>300.90	0.005	30	23
Aflatoxin B2	315.10>259.05	0.005	30	28
	315.10>287.16			25
IS Aflatoxin B2	332.00>303.10	0.005	30	25
Aflatoxin G1	329.09>243.10	0.005	25	26
	329.09>283.00			26
IS Aflatoxin G1	346.00>257.00	0.005	25	26
Aflatoxin G2	331.05>245.05	0.005	25	30
	331.05>257.04			25
IS Aflatoxin G2	348.00>330.00	0.005	25	25
Ocratoxin A	404.20>221.10	0.005	25	30
	404.20>339.10			30
IS Ocratoxin A	424.00>250.00	0.005	25	25
Deoxynivalenol	297.19>231.18	0.037	20	10
	297.19>249.18			10
IS Deoxynivalenol	321.10>263.00	0.039	15	10
Zearalenone	319.20>185.20	0.005	20	19
	319.20>187.20			23
IS Zearalenone	337.00>199.10	0.005	20	19
Fumonisin B1	722.78>334.29	0.005	30	40
	722.78>352.22			35
IS Fumonisin B1	756.10>374.20	0.005	30	40
Fumonisin B2	706.57>318.22	0.005	30	40
	706.57>336.04			40
IS Fumonisin B2	740.20>358.20	0.005	30	36
Toxin T2	484.40>185.10	0.005	10	22
	484.40>215.10			22
IS Toxina T2	508.00>198.10	0.005	25	22

Table S2 - Mycotoxin contamination in the experimental diet of lambs.

DIET	AFB1	AFG1	Aflatoxinas Total	DON	FB1	ZEA	OTA	T-2
Control	ND	ND	-	ND	195	99.0	ND	ND
Mycotoxin	101	125	226	1832	15810	553	ND	ND
Anti-mycotoxin	96.0	137	233	1945	16105	590	ND	ND

ND = Not-detected. Quantification limit: AFB1 and AFG1 = 5.0 ppb/ DON = 100 ppb/FB1 = 100 ppb/ZEA = 20 ppb/ OTA = 5 ppb/ T-2 100 ppb
